# Geometric Feature Characterization of Apple Trees from 3D LiDAR Point Cloud Data

**DOI:** 10.3390/jimaging11010005

**Published:** 2024-12-31

**Authors:** Md Rejaul Karim, Shahriar Ahmed, Md Nasim Reza, Kyu-Ho Lee, Joonjea Sung, Sun-Ok Chung

**Affiliations:** 1 Department of Agricultural Machinery Engineering, Graduate School, Chungnam National University, Daejeon 34134, Republic of Korea; mrkarim@o.cnu.ac.kr (M.R.K.); shahriar@o.cnu.ac.kr (S.A.); reza5575@cnu.ac.kr (M.N.R.); elkyu0927@cnu.ac.kr (K.-H.L.); 2 Department of Smart Agricultural Systems, Graduate School, Chungnam National University, Daejeon 34134, Republic of Korea; 3 FYD Company Ltd., Suwon 16676, Republic of Korea; enature1004@fydkr.com

**Keywords:** smart agriculture, LiDAR sensor, point cloud data, tree canopy volume, tree recognition

## Abstract

The geometric feature characterization of fruit trees plays a role in effective management in orchards. LiDAR (light detection and ranging) technology for object detection enables the rapid and precise evaluation of geometric features. This study aimed to quantify the height, canopy volume, tree spacing, and row spacing in an apple orchard using a three-dimensional (3D) LiDAR sensor. A LiDAR sensor was used to collect 3D point cloud data from the apple orchard. Six samples of apple trees, representing a variety of shapes and sizes, were selected for data collection and validation. Commercial software and the python programming language were utilized to process the collected data. The data processing steps involved data conversion, radius outlier removal, voxel grid downsampling, denoising through filtering and erroneous points, segmentation of the region of interest (ROI), clustering using the density-based spatial clustering (DBSCAN) algorithm, data transformation, and the removal of ground points. Accuracy was assessed by comparing the estimated outputs from the point cloud with the corresponding measured values. The sensor-estimated and measured tree heights were 3.05 ± 0.34 m and 3.13 ± 0.33 m, respectively, with a mean absolute error (MAE) of 0.08 m, a root mean squared error (RMSE) of 0.09 m, a linear coefficient of determination (r^2^) of 0.98, a confidence interval (CI) of −0.14 to −0.02 m, and a high concordance correlation coefficient (CCC) of 0.96, indicating strong agreement and high accuracy. The sensor-estimated and measured canopy volumes were 13.76 ± 2.46 m^3^ and 14.09 ± 2.10 m^3^, respectively, with an MAE of 0.57 m^3^, an RMSE of 0.61 m^3^, an r^2^ value of 0.97, and a CI of −0.92 to 0.26, demonstrating high precision. For tree and row spacing, the sensor-estimated distances and measured distances were 3.04 ± 0.17 and 3.18 ± 0.24 m, and 3.35 ± 0.08 and 3.40 ± 0.05 m, respectively, with RMSE and r^2^ values of 0.12 m and 0.92 for tree spacing, and 0.07 m and 0.94 for row spacing, respectively. The MAE and CI values were 0.09 m, 0.05 m, and −0.18 for tree spacing and 0.01, −0.1, and 0.002 for row spacing, respectively. Although minor differences were observed, the sensor estimates were efficient, though specific measurements require further refinement. The results are based on a limited dataset of six measured values, providing initial insights into geometric feature characterization performance. However, a larger dataset would offer a more reliable accuracy assessment. The small sample size (six apple trees) limits the generalizability of the findings and necessitates caution in interpreting the results. Future studies should incorporate a broader and more diverse dataset to validate and refine the characterization, enhancing management practices in apple orchards.

## 1. Introduction

The apple fruit is one of the dominant fruits among the six primary fruits, namely apples, peaches, grapes, tangerines, persimmons, and pears, which, together, account for about 90% of total fruit production [1]. Apple has the largest contribution, about 25%, which is a significant contribution to the total value in South Korea [1,2]. The Korean orchard industry has faced challenges; in particular, apple production was projected to decrease by 23%. As a result, in South Korean orchard sectors, sensing technology created an importance to increase apple fruit production [2].

Sensing methodology can determine the tree height [3,4,5,6,7,8,9,10,11,12], canopy volume [12,13], row spacing, and tree spacing of orchard fruit trees such as apple trees. This information can be used to improve fruit cultivation and help develop autonomous agricultural machinery for the geometric characterization of fruit trees as well as land characteristic measurements. Fruit trees are crucial to the production of agriculture, and the geometric characterization of fruit trees plays an important role in effective fruit tree management in orchards [14]. The features of fruit trees, such as the height and canopy volume, provide valuable reference data for precise irrigation management [2], effective disease control [12,14,15], and the accurate evaluation of fruit quality [16]. Similarly, row spacing and tree spacing are vital parameters to consider for effective fruit cultivation in an orchard. Characterizing the plant canopy holds significant importance across various agricultural applications [15,16], such as fertilizer and pesticide application to ensure better adjustment of the pesticide dose, irrigation to establish precise crop water need estimation, and crop training to ensure optimal light distribution within the treetops and better fruit quality [12,17].

Digital calipers were used to measure the crown diameter and tree height, as detailed by Morgan et al. [18]. Although the method was valuable, it was time-consuming and destructive. Non-destructive technologies for the geometric characterization of fruit trees, such as thermal imaging, VIS–NIR spectroscopy, multispectral imaging, LiDAR, digital imaging, and hyperspectral imaging, were found to be used in the field [19]. Visible and near-infrared (VIS–NIR) spectrometers were found to be effective and convenient tools for collecting spectral data [20,21]. Various techniques for computing tree canopy dimensions included visible range cameras [22], ultrasonic sensors [23], spectral and infrared sensors [24], and laser sensors [25,26]. Even with considerable efforts to characterize tree canopies, several challenges effectively persisted in implementing these methods in real field conditions. Unpredictable weather conditions are a primary limitation of these methods. The effectiveness of precision management techniques often declines in field conditions due to factors such as variations in illumination, wind direction, wind speed, and vibrations, particularly when using cameras. Inconsistent data from ultrasonic sensors often arise due to the wide dispersion angle of their waves and unpredictable environmental conditions [27]. The challenge in obtaining precise data with spectral and infrared sensors is primarily due to their high sensitivity to natural light and weather variations in outdoor environments [27].

Method validation using measured parameters showed that the geometric features of the fruit trees can be correctly predicted under actual field conditions. However, some differences between sensor-estimated data and measured data readings were noticed in the fruit orchard. These differences were most likely caused by the variability in walking speed during the movement of sensor configuration when data acquisition was being carried out in the apple orchard. LiDAR-derived parameters may be influenced by varying sensor setups, leading to differences in data accuracy related to specific crop characteristics [12,28]. To achieve comprehensive phenotyping, greater attention should be paid to data processing, which can simplify the complexity of plant geometric features and foster new hypotheses [29,30]. Furthermore, there was a need to develop advanced mathematical and statistical approaches as they are vital for analyzing and interpreting complex, dynamic, and multi-temporal geometric features. Even with the use of hyperspectral LiDAR, it is important to recognize that plant characteristics are inherently dynamic and vary at the cellular level and over time.

The canopy volume of fruit trees has been calculated by taking distance measurements with multiple sensors [31]. A commercial software solution was developed to manage field data from ultrasonic sensors, allowing for the measurement of 13 trees’ heights and canopy volumes per minute in citrus groves [32]. Different imaging devices were also utilized to accelerate data acquisition [33,34]. These techniques have facilitated the extension of research from individual leaves to whole orchards, advancing the study of high-throughput phenotypic characteristics. LiDAR is a promising sensor for measuring spatial coordinates and providing reliable location data to describe spatial variability in the geometric features of plants [35,36]. It is widely used for the 3D reconstruction of plants due to its high spatial resolution, low beam divergence, and versatility under various lighting conditions [37,38,39]. LiDAR is an active laser scanning technique for remote sensing which is widely used to characterize tree canopies [40,41]. Numerous studies have explored the use of LiDAR scanning technology for canopy measurements, highlighting its accuracy, high scanning speed, and insensitivity to varying light conditions. These studies consistently demonstrate strong correlations between LiDAR measurements and field observations ranging from 0.85 to 0.95 [42,43,44]. The development of a laser scanning method was pioneered by Wei and Salyani [45] for measuring the height, width, and volume of citrus tree canopies. The study demonstrated an accuracy of 96% in height measurements across three perpendicular dimensions using a LiDAR sensor. In another study, a 270° radial range laser scanning sensor was evaluated for measuring target surfaces with complex shapes and varying sizes [46]. The sensor provided precise X, Y, and Z coordinate data at various travel speeds and detection distances, showing good results. However, ground surface undulation caused significant errors, leading to inaccurate target profile estimation. When estimating the canopy structures, the LiDAR sensor traditionally overlooked the potential impact of errors associated with altitude angles [31]. However, the laser sensing techniques were unaffected by outdoor conditions and offered more precise detection results [25]. Different studies estimated geometric features, such as the canopy volume, using LiDAR sensors [47,48,49,50]. The 3D structure of orchard trees was evaluated, and a computational analysis of plant geometry was conducted using LiDAR sensors, achieving accuracy levels of up to 30% [12,47]. A terrestrial LiDAR scanner was used to estimate the canopy volume with a coefficient of determination (r^2^) of 0.77 [51]. Additionally, a mobile 3D LiDAR mapping technique estimated the canopy volume of apple trees, yielding correlation values of 0.81 and 0.51 for the convex hull and voxel grid methods, respectively, by comparing measured data with sensor-estimated data [48]. For this reason, point cloud data processing and the development of techniques for plant feature characterization should be taken into consideration.

LiDAR data acquisition and processing methods were developed to create 3D tree models and extract canopy volume data [17]. Subsequent research focused on further extracting canopy geometry from 3D point clouds [52]. Using an occupancy grid and connecting outer points are the two main methods for estimating canopy volume by object shape reconstruction, which has broad applications in forestry, agriculture, and environmental sciences [47]. Due to the limitations of ultrasonic sensors, LiDAR sensors became popular for analyzing canopies of various orchard trees, precisely measuring the geometry features of trees and plants [12,53]. Moreover, LiDAR sensors could be considered cost-effective sensors for the rapid supervision of orchard fruit trees since the sensor provides a quick and precise assessment of geometric features without extensive data processing.

Researchers demonstrated that LiDAR effectively characterizes plant morphological features, including the height, canopy volume, and spacing, while offering greater efficiency for large-scale feature estimation [8,12,54]. LiDAR is also used for precise topographic mapping, vegetation structure analysis, and crop height estimation, providing essential information on plant morphological characteristics [55]. Methods and techniques were developed for accurately estimating apple tree volume using commercial LiDAR systems and UAV platforms, coupled with commercial software for manually extracting individual apple trees and processing 3D point clouds [56]. Various pre-processing and processing approaches were used to extract individual tree point clouds, estimate canopy volumes, and generate tree mappings [57]. Accurate measurements of tree and row spacing are essential for precision orchard management [12]. While these parameters are initially determined at the planting stage, they can vary over time due to uneven tree growth, mechanical damage, or changes in management practices. LiDAR technology provides precise data to identify suboptimal spacing early, enabling informed decisions on tree thinning, irrigation, and pest management, which are critical for maintaining orchard productivity. Regular monitoring supports adjustments in management practices to optimize tree health and maximize fruit yield. Berk et al. [50] demonstrated that using a mobile laser scanning (MLS)-type LiDAR for apple tree foliage measurements, combined with the trapezoidal method and commercial software, enabled accurate spacing analysis and canopy volume estimation, with the latter being the most consistent method for digital reconstruction.

Geometric feature characterization is essential for optimizing orchard fruit production, yet limited studies have focused on estimating tree spacing and row spacing using LiDAR technology. This study addressed this gap by applying 3D LiDAR point cloud data to precisely characterize the key geometric features of apple trees, including tree height, canopy volume, tree spacing, and row spacing. Unlike previous research that often emphasized large-scale vegetation mapping or lacked comprehensive validation, this work integrated detailed ground-truth measurements with LiDAR-derived data to ensure high accuracy and reliability. The study introduced small-scale, high-precision measurements targeting individual trees, rigorous postprocessing to reduce noise and enhanced data accuracy, and a robust statistical framework for validation. By addressing orchard-specific parameters, the proposed method provided actionable insights for precision orchard management, contributing to resource optimization, sustainable farming practices, and scalable solutions for similar agricultural systems. Therefore, the objective of this study was to characterize geometric features such as tree height, canopy volume, tree spacing, and row spacing in an apple orchard using 3D LiDAR point cloud data.

## 2. Materials and Methods

### 2.1. Experiment Site and LiDAR Sensor Selection

**Figure 1:**
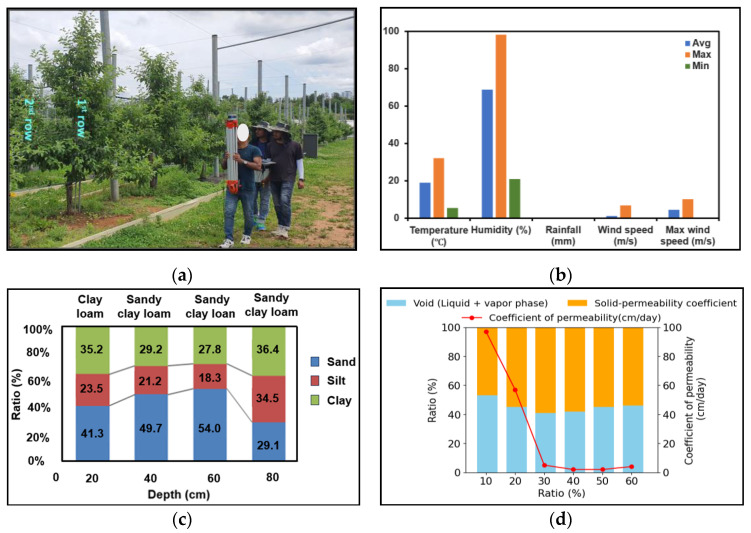
The experiment site. (**a**) The apple orchard; (**b**) the temperature, humidity, rainfall, wind speed, and maximum wind speed during data acquisition in the orchard; (**c**) soil types; and (**d**) soil properties.

The experiment was conducted in an apple orchard located at the National Institute of Horticultural and Herbal Science (NIHHS), Rural Development Administration (RDA), Jeonju, Korea Republic. Figure 1a shows the location of the site. This site was selected for its representative horticultural conditions, providing an ideal setting for testing and validating the use of LiDAR sensors in estimating and analyzing the geometric features of apple trees. Apple trees were planted in two rows, each containing 15 trees. For height and canopy measurements, a total of six trees (three consecutive apple trees from each row), exhibiting different sizes and shapes to represent the variability of the orchard, were selected for intensive data acquisition among the fifteen trees in each row, as shown in Figure 1a. For tree and row spacing measurements, six consecutive apple trees from each row were selected for six iterative distance measurements. The apple variety was Red Rose (*Syzygium jambos*), planted in a spindle shape, and the trees were nine years old. Data collection took place in mid-May 2023. Weather and soil conditions were recorded during the data collection period, as detailed in Figure 1b–d. The orchard soil was loamy, and the weather was sunny with no rainfall. The average temperature during data collection was 19 °C, with a maximum of 36 °C and a minimum of 5.7 °C. Humidity levels averaged 68%, ranging from a maximum of 98% to a minimum of 20%. Wind speeds averaged 5.24 ms^−1^, with a maximum of 10.30 ms^−1^ and a minimum of 1.10 ms^−1^.

A commercial LiDAR (model: VPL−16, Velodyne Lidar, San Jose, CA, USA) was used for this experiment. Table 1 provides the detailed specifications of the LiDAR sensor. The LiDAR has a scanning range of 100 m, low power consumption, a light weight design, a compact size, and dual return capability that make the LiDAR ideal for applications such as autonomous vehicles, robotics, and terrestrial 3D mapping. The sensor has 16 channels and can capture approximately 300,000 points per second. The LiDAR offers a 360° horizontal field of view and a 30° vertical field of view, with a ±15 vertical tilt. Despite having visible rotating parts, the LiDAR is highly resilient and operates effectively over a wide temperature range, providing high-definition and 3D information about the surrounding environment.

**Figure 2:**
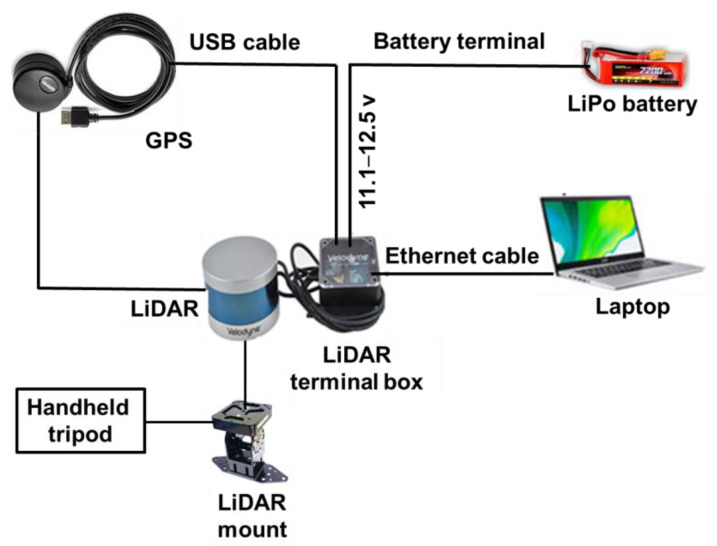
LiDAR sensor setup for data acquisition in this study.

The data acquisition setup for the LiDAR sensor was carefully assembled to ensure precise and effective operation. It included the LiDAR, which served as the primary sensor for capturing 3D spatial data. This was complemented by the LiDAR sensor terminal box for data management, a high-speed laptop computer for real-time processing and analysis, and a 12 V battery to provide power. The LiDAR was mounted on a dedicated plate to ensure stability during operation. Additionally, an external GPS unit (model: GPS18x LVC, Garmin, Martinez, CA, USA) was used for accurate geolocation and data synchronization. Commercial software (model: Veloview, Ver 5.1.0, Kitware, Inc., Clifton Park, NY, USA) was used for data acquisition, which provided tools for visualizing, selecting, and analyzing the data captured by the LiDAR sensor. The software allowed for the visualization of distance measurements as point cloud data and offered customizable color maps for variables such as laser ID, intensity of return, dual return type, azimuth, time, and distance. It also provided options to export data in CSV format as x, y, or z coordinates or point cloud data. Additionally, commercial software and python programming language software were used for integrating and processing 3D point cloud data. The software facilitated further data visualization, analysis, and measurement, as described by Zulkifli et al. [58]. Figure 2 illustrates a schematic diagram of this configuration, detailing how each component was integrated to optimize data collection and system performance.

### 2.2. Sensor Data Acquisition and Processing Procedures

**Figure 3:**
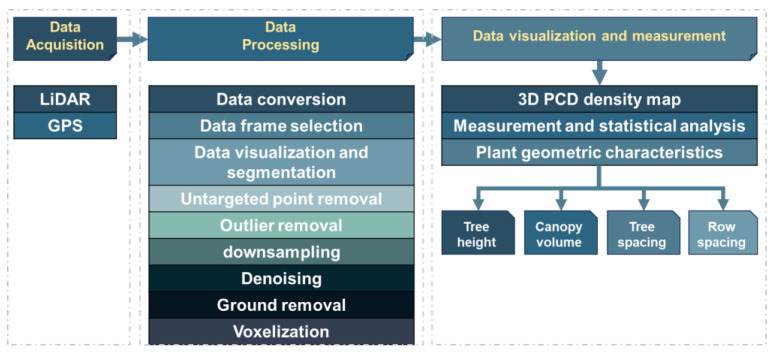
Sensor data acquisition, processing, visualization, and measurement process.

Field data acquisition, processing, and measurement using the LiDAR sensor were divided into three distinct phases: data acquisition (apple tree scanning), data pre-processing, and data analysis. Data acquisition involved scanning the apple trees to capture the necessary information. Following this, data pre-processing was conducted to clean and prepare the raw data for analysis. Finally, data analysis was performed to measure and interpret the results. Figure 3 illustrates a schematic diagram outlining these procedures. The procedure was explained step by step. Data acquisition started with the use of the LiDAR sensor, which captured 3D point cloud data from an apple orchard. The raw point cloud data captured by the LiDAR was stored in a PCAP file format.

The first step in pre-processing was to read this raw data file. In the context of point clouds, data frames in the raw data typically referred to how the point cloud data were organized during capture, processing, and analysis. The step of data frame selection that included each tree involved selecting the data frames from the point cloud data, ensuring that the trees were adequately captured. Then, the coordinates of the point cloud data were transformed using a matrix to adjust the data for further processing in the step of transforming each coordinate of point cloud data. A matrix was used where the transformation was involved in aligning the data with a specific reference frame or correcting for any distortions. The points that were not representing the objects of interest (apple trees) were removed from the data, particularly the ground plane during the removal of the untargeted points (ground plane). Through outlier removal, the outlier points were eliminated as they were significantly different from most of the data, potentially due to noise or errors during data acquisition. The ground sampling process involved sampling the ground data to further refine the dataset by focusing on relevant points. Noise within the point cloud data was reduced to improve the accuracy and clarity of the data, particularly focusing on removing irrelevant data points in the denoising step.

In the ground removal step, the ground points were hidden or excluded from the dataset to focus solely on the trees through hide ground. For the extraction of tree data, data points were selected from trees (region of interest) only without the ground through hiding ground and automatically segmented using python code. After hiding the ground, the dataset contained only the points that represented the trees. The selected points were then used to prepare a 3D point cloud density map, which visually represented the density and distribution of the points that made up the trees for visualization. Finally, using the codes of the Python programming language, the tree metrics such as height, canopy volume, and the spacing between trees and the rows of trees were quantified from the 3D point cloud density map. The map was prepared from the processed data to meet the intended applications.

**Figure 4:**
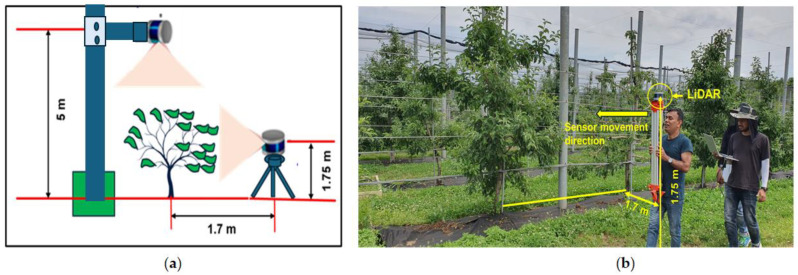
A schematic diagram of the sensor’s position during data acquisition in the apple orchard. (**a**) The sensor’s height from the ground and the distance from the target object (apple tree); (**b**) the movement of the LiDAR sensor during orchard scanning, maintaining a constant distance from the object (apple tree).

The LiDAR was mounted on a tripod and carried together by hand through the center of the apple tree rows while scanning the side view of the trees, maintaining a normal walking speed. The sensor was positioned 1.75 m above ground. Based on the average tree height shown in Figure 4, the distance from the sensor to the centerline of the tree rows was 1.7 m for side view scanning. The field of view (FOV) was designed to cover the entire tree height. The LiDAR was mounted on the tripod, maintaining horizontal alignment for height measurement. There was then a second pass where the lidar was 5 m above ground, and for that, the LiDAR was mounted on an aluminum profile structure to scan the tree structure from the top for canopy volume measurement. The horizontal FOV of the LiDAR was set to 180° for scanning from the side to measure the tree side profile (side view), allowing the differences between the maximum and minimum Z coordinates to determine the apple tree height. The vertical FOV of the LiDAR was also set to 180° for scanning the top profile of the tree (top view) to measure the canopy volume. For measuring row spacing, the FOV was set to 360° to scan both the left- and right-side tree profiles while moving the LiDAR through the center of two rows of apple trees. Point cloud (PCD) data were acquired for six consecutive apple trees. Figure 4 illustrates the data acquisition procedure in the apple orchard. The LiDAR sensor used in the experiment had a scanning speed of 23 frames per second. Height, canopy volume, tree spacing, and row spacing were estimated to compare the measured results with sensor-estimated results.

In this study, the LiDAR was mounted on a tripod for side-view scanning, where a handheld aluminum structure was used to scan the tree structure from the top, allowing for the dynamic scanning of individual trees in the orchard. The approach offered flexibility in accessing various angles of the canopy without the need for a fixed scanner. While using a fixed scanner could be a limitation for larger areas, the handheld scanning method allowed efficient and scalable data capture, even in extensive orchard environments. The ability to move through the orchard helped maintain control over the scanning process to achieve a suitable scan of a broader area with varying canopy structures. Additionally, utilizing a walking path, enabled adjustments to the scanning angle to capture data from different perspectives, which helped to address the challenges associated with the larger orchard area.

**Figure 5:**
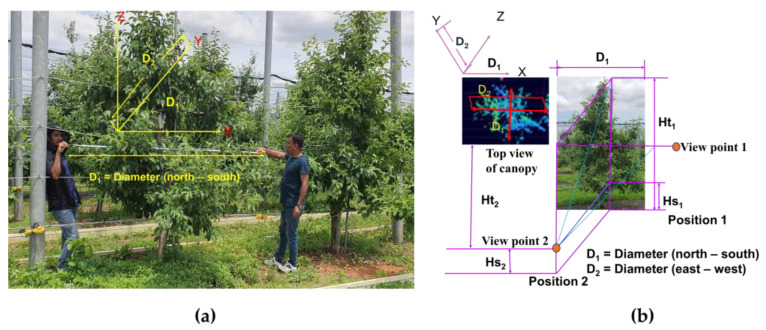
Measured canopy volume of apple tree: (**a**) diameter measurement; (**b**) measurement of measured canopy volume according to Equation (1).

During LiDAR data acquisition, tree height, tree spacing, and row spacing were manually measured using a measuring tape, and the data were recorded. Multiple measurements were taken for six trees and averaged all collected data. The standard deviation was calculated to ensure accuracy. Figure 5 illustrates the manual measurement procedure for the canopy volume.

In manual measurement, the canopy volume profiles were triangular, truncated rectangular, or rectangular rather than the assumed ellipsoid shape. The manual canopy volume (CV_m_) was calculated using Equation (1) [59]:

(1) $${CV}_{m}=\frac{1}{6}{\pi D}_{1}\times D_{2}\times \frac{1}{2}(\left({Ht}_{1}-{Hs}_{1}\right)+\left({Ht}_{2}-{Hs}_{2}\right))$$

where D_1_ and D_2_ are the crown diameters in the north to south direction and east to west direction, and ${Hs}_{1}$ and ${Hs}_{2}$ are the height between tree trunk starting point and ground measured from positions 1 and 2, respectively. ${Ht}_{1}$ is the total height of the tree from the ground to the top of the tree measured with respect to position 1, and ${Ht}_{2}$ is the height of the center point of the tree trunk from viewpoint 2 measured from position 2.

From two perpendicular positions, the height of the tree was measured, and the crown diameters were labeled as D_1_ and D_2_, respectively, as shown in Figure 5a. To obtain the diameters and heights of canopy geometry from two measuring positions, the measuring posts were used as shown in Figure 5b. From the first measuring position, a measuring post was vertically placed at the canopy center of the apple tree, and we took measurements of the outer parts of the canopy (${Ht}_{1}$ and ${Hs}_{1}$). Then, we took measurements from position 2, which was located 1.7 m away from the measuring post and was also vertically placed at the canopy center. The measurements of the outer parts of the canopy were recorded as ${Ht}_{2}$ and ${Hs}_{2}$. Afterward, from measuring position 1, the measuring post was horizontally placed on the ground from where the canopy surface (tree trunk) started. The measuring post was extended from the two outermost points of the crown in that direction, and the canopy width (D_1_) was measured from position 2. These steps were then repeated at a position of 90° from the initial one.

The point cloud data were processed after data collection. The scanning process required an interface to display the point cloud data and save information from the LiDAR sensor. Commercial software (model: VeloView, Ver: 5.1.0, Kitware, Inc., Clifton Park, NY, USA) was used to read the data from the scanning process before preparing the 3D point cloud density map. This map was then used to compare the original images of the apple trees with the intensity information collected from the apple orchard. Commercial software and the Python programming language were used to perform real−time visualization and processing of live captured 3D LiDAR data, reading, and saving the raw data as packet capture (pcap) format into the notebook.

The data were visualized, and the point cloud data were edited. The data processing steps involved outlier removal, downsampling, denoising, segmentation of the region of interest (ROI), clustering, data transformation, and ground removal. An established coordinate system, known as the right-hand rule for 3D cartesian coordinates, was followed for this study, where the tree row was parallel to the X-axis, perpendicular to the Y-axis used to define the width or cross−row distance, and the tree trunk pointed vertically upward along the Z-axis. During LiDAR data collection, the sensor was maintained in vertical alignment with the ground, resulting in the acquired point cloud data being orientated at a 90° counterclockwise angle. To correct this orientation, a two-phase transformation was applied within the defined coordinate system.

In the initial phase, each coordinate within the dataset underwent a counterclockwise rotation with respect to the Z-axis. In the next phase, the resulting coordinates were rotated clockwise with respect to the Y-axis, according to Equations (2) and (3), respectively. This systematic transformation facilitated the reorientation of the data into a more suitable configuration for subsequent analysis and interpretation.

(2) $$R_{z}\;(\theta =90{}^{\circ})=\left[\begin{array}{cccc} \cos\theta & -\sin\theta & 0 & 0\\ \sin\theta & \cos\theta & 0 & 0\\ 0 & 0 & 1 & 0\\ 0 & 0 & 0 & 1 \end{array}\right]$$

A 90° clockwise rotation around the Y-axis:

(3) $$R_{y}\;(\phi =90{}^{\circ})=\left[\begin{array}{cccc} \cos\phi & 0 & -\sin\phi & 0\\ 0 & 1 & 0 & 0\\ \sin\phi & 0 & \cos\phi & 0\\ 0 & 0 & 0 & 1 \end{array}\right]$$

**Figure 6:**
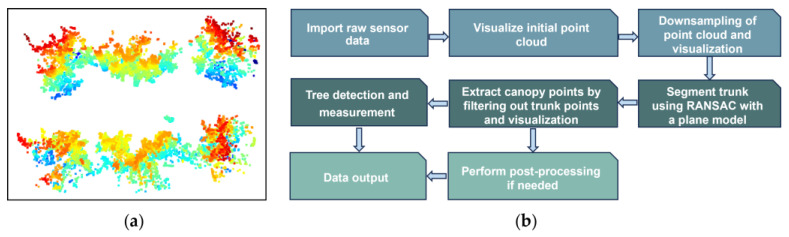
Feature extraction algorithm of trees. (**a**) Targeted points’ segmentation carried out by specifying ROI; (**b**) schematic diagram of algorithm.

The transformed point cloud data included points from trees in different rows. To isolate the targeted points from an individual tree, the region of interest (ROI) was selected. Figure 6b shows the feature extraction algorithm. Targeted points were segmented by specifying the ROI and using Kd-tree means search algorithm. The Kd-tree (k-dimensional tree) organizes points in a k-dimensional space for efficient searching. It was constructed by recursive partitioning the dataset along different dimensions; in particular, the first partition was along the X-axis, the second was along the Y-axis, and the third was along the Z-axis. It facilitated efficient searching for points within the ROI, such as nearest neighbor search and range search. The algorithm quickly located the nearest neighbors of the given point by traversing the tree, reducing the number of points that need to be examined. Additionally, for the given ROI, the Kd-tree rapidly identified all points within that region by only exploring relevant parts of the tree, significantly speeding up the search process. The Kd-tree was employed to segment the point cloud by identifying and isolating points that fell within the ROI. This allowed for the extraction of points corresponding to the individual tree from the overall dataset.

The region of interest (ROI) for canopy estimation in the orchard was defined along the X, Y, and Z axes to capture the specific spatial extent of the trees such as tree dimension, field layout, data coverage, empirical testing, uniformity of tree characteristics, alignment with orchard rows, precision in canopy extraction, and adaptability. The size of trees within the orchard was a critical consideration. The ROI values were chosen to encompass the typical height, width, and row length of the trees. By analyzing preliminary data or performing exploratory measurements, the boundaries were established to ensure the entire canopy of each tree, including the extremity, was within the ROI. The layout of the orchard, including the spacing between trees and rows, influenced the ROI. The X-axis and Y-axis values were set to align with the expected positions of the trees in their respective rows, while the Z-axis values were set according to the expected height and ground clearance of the canopies. The ranges along each axis were set to include all relevant points captured during the scanning process, ensuring that no part of the tree canopy was left out. The boundaries were refined through trial and error, adjusting the ROI according to initial results to optimize the inclusion of the entire canopy while minimizing extraneous points. Along the X-axis, the range was chosen to capture the full width of the canopy of a single tree, including some buffer space on either side to account for slight variations in tree shape and positioning. The Y-axis range was set to encompass the entire length of the tree canopy along the row, from one end to the other. This range ensured that the full canopy, as well as the tree trunk, was captured within the ROI. It also provided a buffer to include points slightly beyond the typical tree length to account for variations in growth and structure. Similarly, the Z-axis range was selected to cover the height of the tree from the base (slightly below the ground level) to the top of the canopy. The inclusion of points below ground level ensured that any points mistakenly attributed to the canopy due to noise or errors in data collection were still captured. The upper limit captured the full height of the canopy, ensuring that no part of the structure of the tree was excluded. The extracted points also included the ground plane at the base of the targeted tree, which needed to be removed before isolating the canopy data. To fit the plane in the 3D point cloud, the random sample consensus (RANSAC) algorithm was used, which is an iterative method designed for robustly fitting mathematical models in the presence of numerous data outliers. The RANSAC algorithm was particularly effective in the experiment for separating ground plane points from tree canopy points, treating the ground plane points as outliers.

### 2.3. Tree Geometric Feature Characterization

#### 2.3.1. Tree Height and Canopy Volume

**Figure 7:**
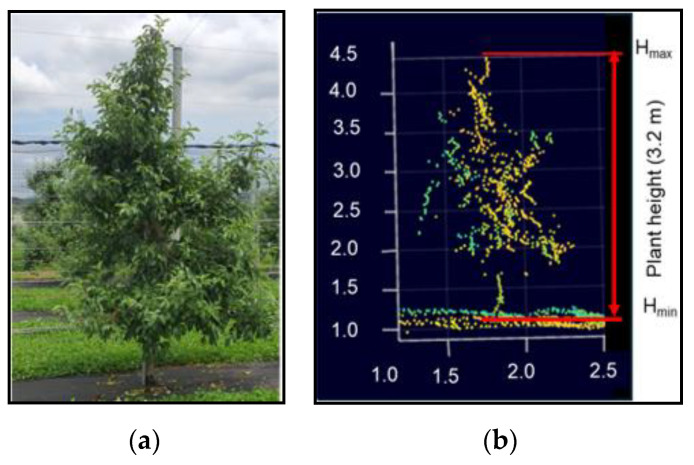
Tree height measurement using LiDAR: (**a**) real view of apple tree; (**b**) LiDAR-estimated tree height.

The python code was used to create a 3D point cloud density map for visualizing and measuring tree features. Following data processing and the generation of 3D point cloud density map, the maximum value of the cloud point ${(H}_{max})$ and the minimum value of the cloud point ${(H}_{min})$ were determined, as shown in Figure 7.

The raw data consisted of packets of information that included the 3D coordinates (x, y, and z) for each point detected by the LiDAR sensor. The data were processed to filter out noise or erroneous points that could result from sensor errors or environmental factors. A technique such as Radius Outlier Removal was applied for removing the outliers. In the context of height measurement of trees, the primary focus was on the values of the vertical axis (z-axis), which represented the height of the points. The maximum height ${(H}_{max})$ was determined by identifying the point in the point cloud with the highest z-coordinate values. This corresponded to the highest point detected by the LiDAR sensor, typically at the top of the tree canopy. The minimum height ${(H}_{min})$ was found by identifying the point with the lowest z-coordinate values. This corresponded to the lowest point detected, often near the ground level or the base of the tree. The tree height was then calculated by subtracting the minimum value from the maximum value, according to Equation (4), as follows:

(4) $$\mathrm{Tree}\;\mathrm{height},H_{\mathrm{tree}}=H_{\max}-H_{\min}$$

where $H_{\max}$ is the maximum height, and $H_{\min}$ is the minimum height.

**Figure 8:**
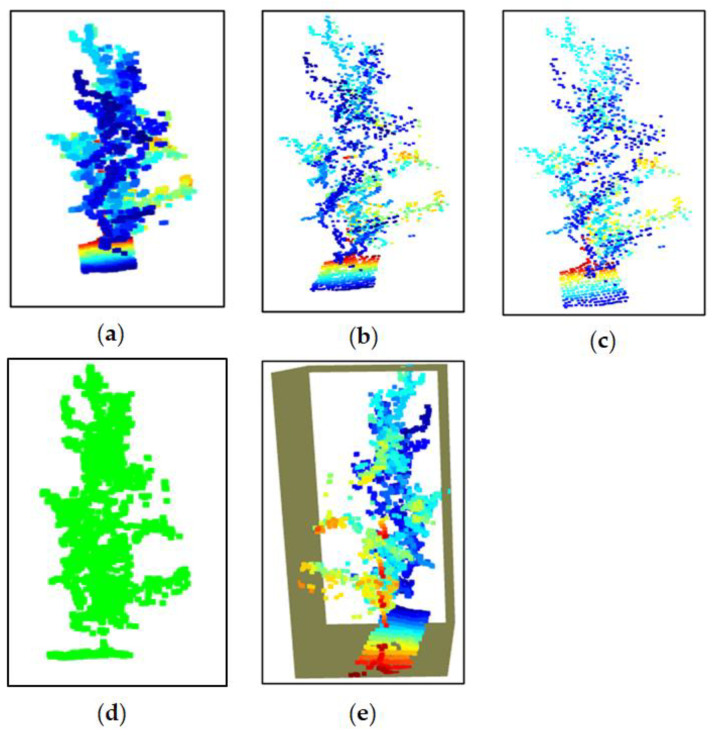
Three-dimensional point cloud density map and bounding box used for canopy volume measurement: (**a**) 3D point cloud; (**b**) downsampling; (**c**) denoising; (**d**) outlier removal; (**e**) canopy volume estimation using bounding box.

**Figure 9:**
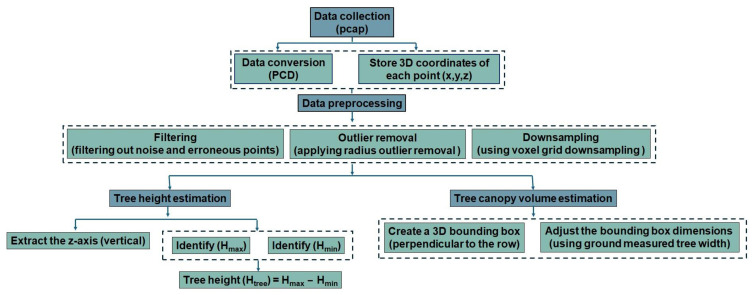
Flow diagram for estimating tree height and canopy volume.

The tree canopy volume was calculated from the 3D point cloud density map using a bounding box. When using a LiDAR sensor from one side of the tree row, the point cloud captured the front-facing side of the tree canopy. In such a case, the bounding box based on the available point cloud only represented the visible portion of the canopy. This could lead to an underestimation of the canopy volume. Measurements of the tree width (perpendicular to the row) were available from manual ground measurements, and based on the width, the bounding box dimensions were adjusted. Figure 8 shows the 3D point cloud density map and the bounding box used for measuring the canopy volume. The point cloud represented the external surface of the apple tree captured in 3D space, particularly in the apple orchard. The downsampling process reduced the number of points in the point cloud while maintaining the overall structure. The voxel grid method through voxel downsampling, which was used in the data processing, reduced the number of points by grouping them into cubes (voxels) and replacing them with a single point. This made the data less dense and more manageable, which is important for large datasets. Point clouds contained noise, which were points that did not belong to the measured surface of the object. The radius outlier removal technique was applied to remove points that had fewer neighbors within a certain radius and helped to clean up the data. Outlier points are the points that do not conform to the expected pattern, often caused by noise or errors in data acquisition. Removing outliers is essential for accurate modeling. In the code, for radius-based outlier removal, points that had fewer neighbors within a specified radius were considered as outliers in data processing and removed from the dataset. To estimate the canopy volume of the apple tree, a bounding box was generated around the point clouds. The bounding box was essentially the smallest box that contained the entire point cloud of the canopy. The volume of the bounding box gave an estimate of the volume of the canopy. This refined the irregular shape of the tree. In this data processing step, the bounding box was generated based on the extents of the point cloud determining the minimum and maximum x, y, and z coordinates to create a rectangular prism. Figure 9 shows a summary of the estimation process of the tree height and canopy volume to make the description more understandable.

**Figure 10:**
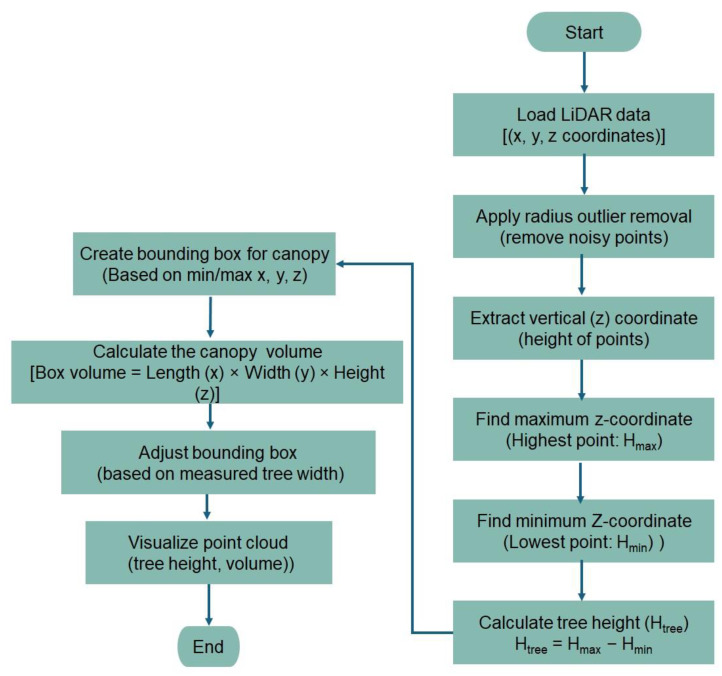
Algorithm used for estimating tree height and canopy volume.

Raw LiDAR point cloud data were captured from the apple orchard and stored the 3D coordinates (x, y, and z) for each detected point. After that, noise and erroneous points caused by sensor errors or environmental factors were filtered out. Radius outlier removal technique (number of points 32 and radius of 0.01) was applied to remove points with fewer neighbors within a specified radius. Voxel grid downsampling (voxel size of 0.01) was used to reduce point cloud density while preserving overall structure. The z-axis (vertical) values were extracted from the processed point cloud data, and the highest detected point the maximum z-value (Hmax) as well as the lowest detected point (often ground level) were identified to calculate the tree height using Equation (4). For canopy volume estimation using sensor, a 3D bounding box was created around the visible portion of the canopy in the point cloud. The bounding box dimensions were adjusted using ground-measured tree width perpendicular to the row. Thus, the bounding box volume was calculated to estimate the canopy volume. A 3D point cloud density map was generated for visualizing and measuring tree features, and the map was used for detailed tree height and canopy analysis. Figure 10 shows the algorithm used for estimating the tree height and canopy volume from point cloud data.

#### 2.3.2. Row Spacing and Tree Spacing

Tree and row spacing were initially fixed when the trees were planted. But it had to be justified that they might change over time due to growth, environmental factors, or even errors during planting. Trees might have uneven growth patterns that could lead to unanticipated deviations in spacing. Regularly measuring and monitoring tree and row spacing with LiDAR provides an opportunity to assess these changes more accurately, allowing farmers to optimize spacing for maximum yield and proper air circulation. Monitoring tree and row spacing could contribute significantly to the productivity of an orchard. If tree or row spacing is found to be suboptimal, plant health, light exposure, and overall growth may be affected. By using LiDAR, farmers can detect small changes in spacing and adjust the growth patterns of the trees accordingly. If trees were too closely spaced, they might compete for resources such as nutrients, water, and sunlight. Adjusting spacing based on accurate data could ensure that each tree received optimal conditions for growth, leading to higher fruit yield and improved quality. Tree and row spacing measurements could also be integrated with other agronomic data, such as soil conditions, water usage, and pest management, to improve the overall efficiency of orchard management. Accurate spacing measurements could help optimize the use of machinery for pruning, harvesting, and spraying, which might help to make the orchards more productive.

**Figure 11:**
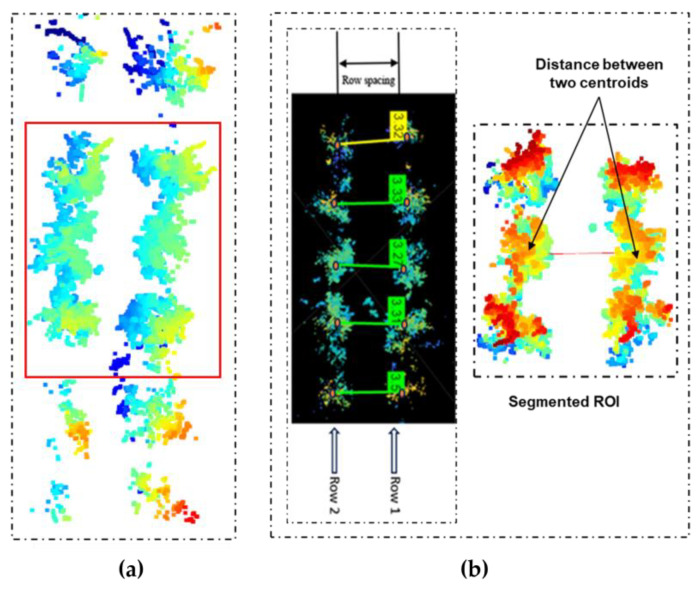
Three-dimensional point cloud density map of apple trees: (**a**) visualization of tree rows and segmented region of interest; (**b**) measurement of row spacing using distance measurement between centroids of two trees in two rows apart from each other.

After preprocessing and generating the 3D point density map, as shown in Figure 11a, the row spacing between consecutive trees was determined by calculating the center-to-center distance, as shown in Figure 11b, using python code. The tree spacing was measured by calculating the center-to-center distance between tress in each row, as shown in Figure 11.

The point cloud data were first clustered using the DBSCAN algorithm. DBSCAN (Density-Based Spatial Clustering of Applications with Noise) identified clusters of closely packed points (representing tree rows) based on their proximity in the x-y plane (such as ignoring the z-coordinate, which typically represents height). Each identified cluster represented a row of trees, and noise points were ignored. After clustering, the points that belonged to the same cluster (row) were grouped together. The centroid (or center point) of each row and each tree in a row were calculated, and the centroid was the average position of all the points in a cluster (tree and row). The centroid was calculated according to Equation (5):

(5) $$C_{x,y,z}=\frac{1}{n}\sum_{i=1}^{n}x_{i},y_{i},z_{i}$$

where n is the number of points in a row, and each point is represented as x_i_, y_i_, or z_i_; i ranges from 1 to n. After calculating the centroid for each row, those centroids were treated as the central points of the tree rows. Lines were then drawn between those centroids to represent the alignment of tree rows.

**Figure 12:**
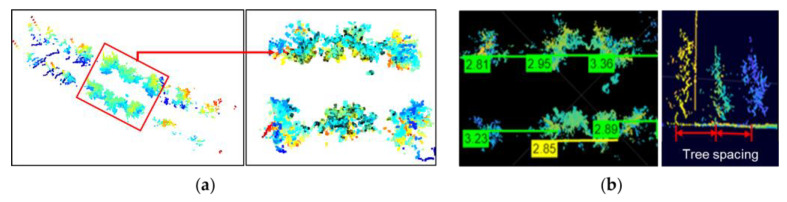
Three-dimensional point cloud density map of apple trees: (**a**) visualization of tree rows and segmentation of region of interest of trees for spacing measurement; (**b**) tree spacing measurement using distance measurement between centroids of two trees in same rows.

**Figure 13:**
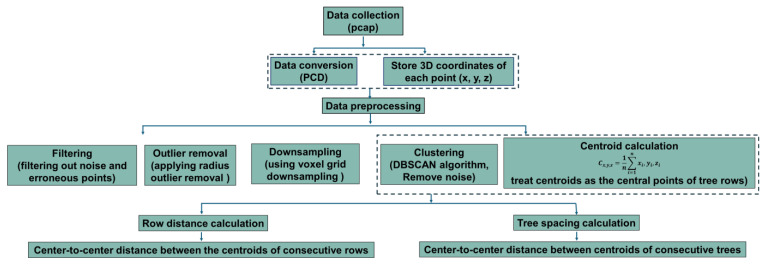
Flow diagram for estimating tree spacing and row spacing.

**Figure 14:**
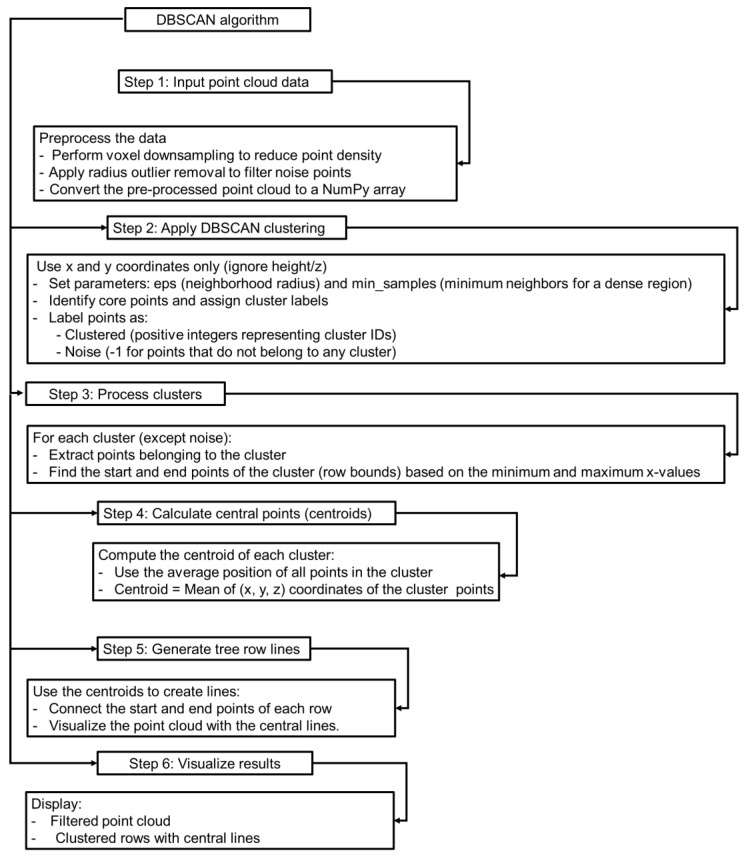
Clustering algorithm used for estimating tree and row distance.

Figure 12 shows a summary of the estimation process of tree spacing and canopy volume to make the description more understandable. Figure 13 and Figure 14 show the algorithm and measurement procedures of row spacing and tree spacing estimation.

Algorithms used in tree spacing and row distance measurement and the errors raised during the measurement were explained step by step. During data pre-processing, radius outlier removal was applied to filter out points that were isolated or significantly distant from their neighbors within a defined radius. In this algorithm, the errors were the incorrect removal of valid points, particularly near the edges of dense clusters, and sensitivity to radius size, where a radius that was too small might remove valid points, while a radius that was too large might retain noise. The algorithm in voxel grid downsampling divided the space into a 3D grid of voxels and replaces all points within each voxel with their centroid, and the errors were the loss of fine details, especially for small features like narrow branches, and the misrepresentation of point density when the voxel size was too large. During clustering, the DBSCAN algorithm was applied, making groups of points based on density and proximity. Points in densely packed areas were classified as clusters, while others were considered as noise. This step performed poorly with non-uniform spaced data and depended on parameters (eps for neighborhood radius and minPts for minimum points), where incorrect values could lead to over-clustering or missed clusters. Moreover, valid points were misclassified as noise where/when the density thresholds were too high. During centroid calculation, the algorithm calculates the arithmetic mean of all points in a cluster to determine its central location according to Equation (6). In this step, the errors were the outliers within a cluster, which skewed the centroid calculation, and the uneven distribution of points, which led to inaccurate centrality. At the time of row and tree spacing calculation, the used algorithm measured the Euclidean distance between centroids of consecutive rows or trees using Equation (6), where possible errors might arise as variability in tree shapes or inconsistent clustering may affect distance accuracy. Overlapping tree canopies might distort row alignment detection in the algorithm used in this step. In visualization algorithm, potential errors might create misalignment of visualized rows due to errors in centroid detection, where visual artifacts might be caused by noise or incorrect clustering. In that case, using adaptive parameter optimization or a pre-analysis of the point density could improve results. Moreover, filtering techniques might remove important features. In this regard, iterative parameter adjustments and validation against ground truth could minimize errors. Also, aligning clusters with known geometries or ground-truth data could validate spacing accuracy. By detailing these algorithms and their potential sources of error, this study ensured transparency and provided a foundation for improving accuracy in future implementations.

(6) $$d=\sqrt{\left\{({x_{2}-x_{1})}^{2}+{\left(y_{2}-y_{1}\right)}^{2}\right\}}$$

### 2.4. Statistical and Analytical Procedures

The geometric features, including tree height, canopy volume, row distance, and tree spacing, were compared between measured and sensor estimated data through linear regression analysis. For better demonstration and understanding of the estimation of geometric features of trees, the mean error calculation was performed. The mean error in tree feature estimation better reflected the accuracy of the estimation method and accounted for the variability between individual trees of different heights, canopy volumes, tree spacing, and row spacing. Statistical analysis was performed using python programming language. To assess the accuracy of the developed data processing algorithm, coefficient of determination (r^2^), root mean squared error (RMSE), mean absolute error (MAE), bias, confidence interval (CI) with 95% confidence for the mean difference, standard deviation ($s_{d}$), the concordance correlation coefficient (CCC), and t-test statistic were calculated using Equations (7) to (15), respectively, as follows:

(7) $$r^{2}=1-\frac{\sum_{i=1}^{n}{\left.(y_{i}-y_{a}\right)}^{2}}{\sum_{i=1}^{n}{\left(y_{i}-\bar{y}\right)}^{2}}$$

(8) $$RMSE=\sqrt{\frac{1}{n}\sum_{i=1}^{n}{\left(y_{i}-y_{a}\right)}^{2}}$$

(9) $$\mathrm{MAE}=\frac{1}{n}\sum_{i=1}^{n}\left|x_{i}-y_{i}\right|$$

(10) $$\mathrm{Bias}=\frac{1}{n}\sum_{i=1}^{n}\left(x_{i}-y_{i}\right)$$

(11) $$\mathrm{CI}=\bar{d}\pm t^{*}.\frac{S_{d}}{\sqrt{n}}$$

(12) $$\bar{d}=\frac{1}{n}\sum_{i=1}^{n}{(x}_{i}-y_{i})$$

(13) $$s_{d}=\sqrt{(\frac{1}{n-1}\sum_{i=1}^{n}{\left(d_{i}-\bar{d}\right)}^{2})}$$

(14) $$\mathrm{CCC}=\frac{2\rho \sigma_{x}\sigma_{y}}{{\sigma_{x}}^{2}+{\sigma_{y}}^{2}+({{µ}_{x-{µ}_{y}})}^{2}}$$

(15) $$t=\frac{\bar{d}}{\frac{s_{d}}{\sqrt{n}}}$$

where $y_{i}$ and $y_{a}$ are the measured and sensor-estimated values, respectively, and $\bar{y}$ is the average of the sensor estimated values. $x_{i}$ and $y_{i}$ are the sensor-estimated values for i^th^ observations. n is the number of observations. Bias indicates whether the sensor estimated values consistently overestimated (>0) or underestimated (<0) the measured values. $\bar{d}$ is the mean difference (bias). $s_{d}$ indicates standard deviation of the differences $\left(d_{i}=x_{i}-y_{i}\right)$, and $t^{*}$ is the critical value of t-distribution with n−1 degrees of freedom at the desired confidence level (95%). ρ is the Pearson correlation coefficient between x (sensor estimated) and y (measured), and $\sigma_{x}$ and $\sigma_{y}$ are the standard deviations of x and y, respectively. ${µ}_{x}$ and ${µ}_{y}$ are the means of x and y, respectively. CCC measures the agreement between the sensor-estimated and measured datasets, combining precision (correlation) and accuracy (bias). The *p*-value was determined by comparing the calculated t-statistic with a t-distribution table or function for n−1 degrees of freedom.

## 3. Results

The features of the apple trees, including the height, canopy volume, row spacing, and tree spacing, were measured from the field using a LiDAR sensor. The measurement was also carried out in field conditions. The mean value, mean absolute error (MAE), standard deviation (S_d_), root mean square error (RMSE), coefficient of determination (r^2^), bias (mean of the differences), concordance correlation coefficient (CCC), confidence interval (CI) for the mean difference, t-statistic, and *p*-value were summarized from both the measured and sensor estimated data in Table 2.

The statistical parameters for tree height, canopy volume, tree spacing, and row spacing measured by the sensor compared with the ground truth data are presented in Table 2. The results show that for the tree height, the mean sensor estimated value of 3.05 m is slightly lower than the measured value of 3.13 m, with a small bias of −0.08 m. The high accuracy of the sensor was supported by a strong correlation between the r^2^ value of 0.98 and a high CCC value of 0.96, indicating a reliable measurement. The RMSE of 0.09 m and the MAE of 0.08 m confirm the ability of a sensor to accurately estimate the tree height. The t-statistic of −3.49 and a *p*-value of 0.02 indicate a statistically significant difference between the sensor estimated and measured values, suggesting that the sensor consistently underestimated the tree height. The 95% CI, ranging from −0.14 to −0.02 m, confirmed this underestimation.

For the canopy volume, the mean sensor-estimated value (13.76 m^3^) is slightly lower than the measured value of 14.09 m^3^, with a bias of −0.33 m^3^. Although the r^2^ value of 0.97 indicates a strong correlation, the lower CCC value of 0.84 and the higher RMSE of 0.61 m^3^ suggest a moderate degree of error. The t-statistic of −1.44 and the *p*-value of 0.21 indicate a statistically non-significant difference between the sensor-estimated and measured canopy volumes, but the sensor slightly underestimated the volume.

Regarding tree spacing, the sensor estimated value of 3.04 m is close to the measured value of 3.18 m, with a bias of −0.09 m. The high r^2^ value of 0.92 and CCC of 0.84, along with the low MAE of 0.09 m, suggest an accurate measurement. The RMSE of 0.12 m and the t-statistic of −2.19 with a *p*-value of 0.08 indicate a small but statistically significant difference between the sensor-estimated and measured values, with the sensor underestimating the tree spacing.

For row spacing, the sensor-estimated value of 3.35 m is slightly lower than the measured value of 3.41 m, with a bias of −0.05 m. The r^2^ value of 0.94 and CCC value of 0.61, along with the low RMSE of 0.07 m and the MAE of 0.05 m, suggest that the sensor performed well in estimating row spacing. However, the t-statistic of −2.47 and the *p*-value of 0.06 indicate a marginally significant difference, with the sensor slightly underestimating row spacing.

**Figure 15:**
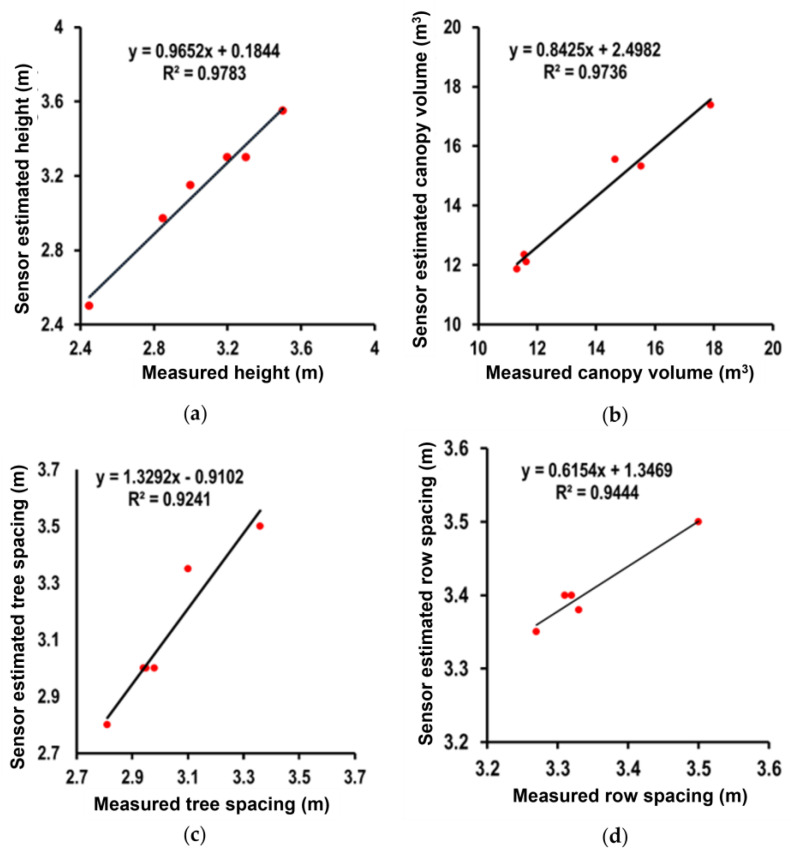
Correlation between measured and sensor estimated results: (**a**) tree height; (**b**) canopy volume; (**c**) tree spacing; (**d**) row spacing.

**Figure 16:**
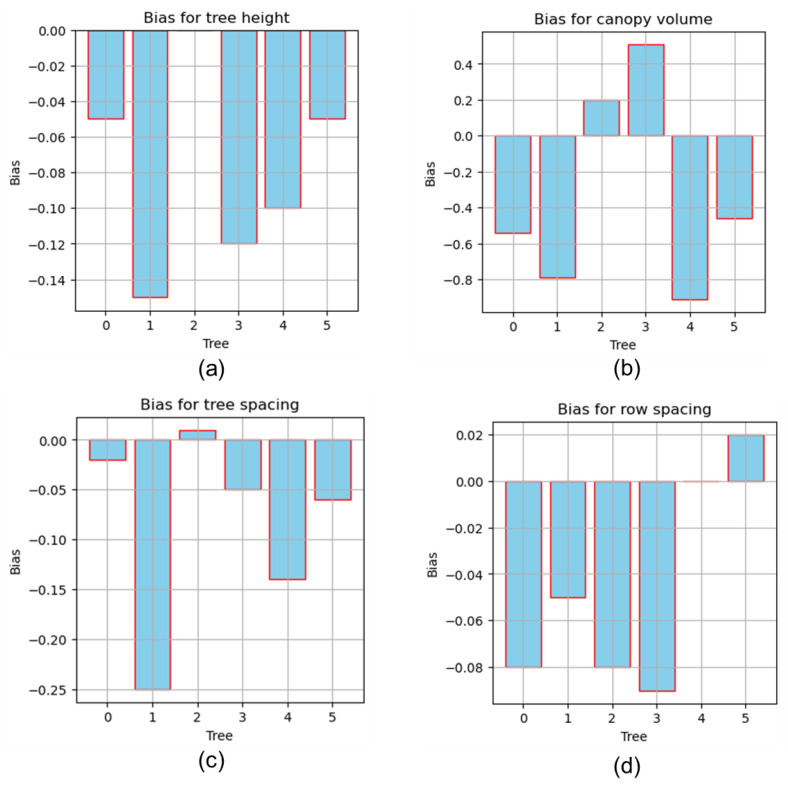
Reliability between measured and sensor-estimated results: (**a**) tree height; (**b**) canopy volume; (**c**) tree spacing; (**d**) row spacing.

**Figure 17:**
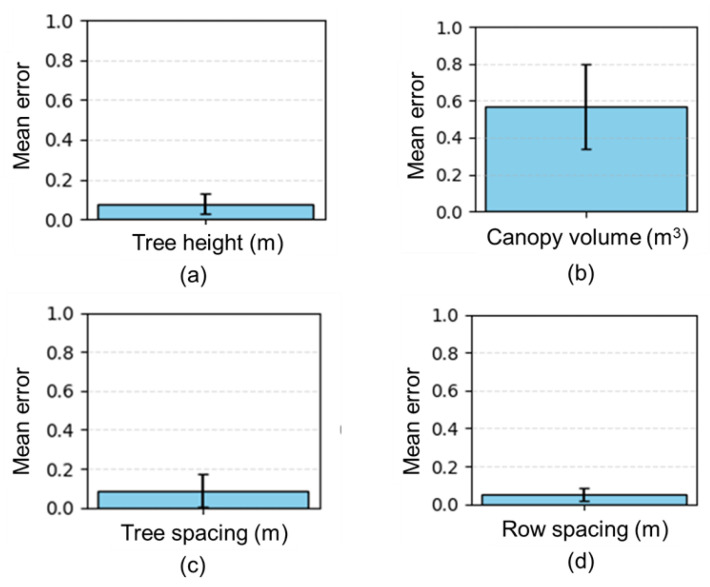
Mean absolute error: (**a**) tree height; (**b**) canopy volume; (**c**) tree spacing; (**d**) row spacing.

Figure 15a, Figure 16a and Figure 17a illustrate the correlation outcomes between the measured and LiDAR estimated values for tree height. The measured average tree height was 3.13 ± 0.33 m, with a minimum value of 2.5 m and a maximum of 3.55 m. In comparison, the LiDAR sensor estimated the average tree height as 3.05 ± 0.34 m, with a minimum value of 2.45 m and a maximum of 3.5 m. The slight difference between the measured and LiDAR estimated values shows that the sensor slightly underestimated the tree height on average. The high correlation coefficient of r^2^ was 0.98 between the two datasets, confirming a strong agreement, indicating that the LiDAR sensor provided a highly reliable estimate for tree height.

Figure 15b, Figure 16b and Figure 17b show the correlation outcomes between the measured and LiDAR-estimated values for canopy volume. The measured average canopy volume was 14.09 ± 2.10 m^3^, with a minimum of 11.86 m^3^ and a maximum of 17.38 m^3^. For the LiDAR estimates, the average canopy volume was 13.76 ± 2.46 m^3^, ranging from 11.32 m^3^ to 17.89 m^3^. The LiDAR sensor generally underestimated the canopy volume compared to the measured values. The r^2^ value of 0.97 indicates a strong correlation between the measured and LiDAR estimated volumes, demonstrating that the LiDAR sensor was accurate in estimating the canopy volume, although with some slight underestimation.

Similarly, Figure 15c, Figure 16c and Figure 17c exhibit the correlation outcomes between the measured and LiDAR estimated values for tree spacing. The average measured tree spacing was 3.18 ± 0.24 m, with a range from 2.8 m to 3.35 m. In comparison, the LiDAR-estimated tree spacing averaged 3.04 ± 0.17 m, with values ranging from 2.81 m to 3.36 m. The LiDAR estimates show a slight underestimation for tree spacing, with an r^2^ value of 0.92. This indicates that while the LiDAR sensor provided a relatively accurate estimate of tree spacing, this exhibited slightly more variability compared to the measurements for tree height or canopy volume.

Figure 15d, Figure 16d and Figure 17d demonstrate the correlation outcomes between the measured and LiDAR estimated values for row spacing. The measured row spacing averaged 3.41 ± 0.05 m, with a range of 3.28 m to 3.5 m. In comparison, the LiDAR sensor estimated an average row spacing of 3.35 ± 0.08 m, with values ranging from 3.27 m to 3.5 m. The LiDAR sensor showed a slight underestimation for row spacing, with an r^2^ value of 0.94, indicating a strong correlation between the sensor estimated and measured values. This suggests that the LiDAR sensor demonstrated high accuracy in estimating row spacing, although a small bias was observed.

## 4. Discussion

For tree height estimation, the sensor exhibited a statistically significant difference compared to the measured values, with a *p*-value of 0.0174 (*p* < 0.05). The confidence interval (CI) of −0.136 to −0.021 m, excluding zero, further validated this significance. On average, the sensor underestimated the tree height by 0.078 m, as indicated by the negative bias. The CI indicated that the mean difference between the sensor-estimated and measured values ranged from 0.021 to 0.136 m with 95% confidence. Despite this minor bias, the sensor demonstrated strong reliability, achieving a concordance correlation coefficient (CCC) of 0.96 and a mean absolute error (MAE) of 0.08 m, signifying high accuracy and agreement. These findings highlight the method’s robustness for tree height estimation, with minor deviations likely caused by environmental variables or sensor adjustments. Previous studies have demonstrated the efficiency of LiDAR in estimating the tree height and other morphological characteristics. Park et al. [60] utilized 3D LiDAR point clouds to estimate sorghum morphological phenotypes, achieving an r^2^ value of 0.83 for the plant height, with high accuracy in other parameters. This was accomplished through voxel grid downsampling and the RANSAC algorithm for outlier removal. Similarly, Lee et al. [61] reported a tree detection accuracy of 95% and tree height estimation accuracy exceeding 0.8 m, consistent with the high r^2^ values found in this study. These findings highlight the precision of LiDAR in height measurement and further support the outcomes of the present study.

Several previous studies of other competitors provided baseline accuracies for LiDAR estimated tree height [3,4,9] focused on forestry or generalized applications, while this study achieved comparable accuracy for apple trees, with an MAE of 0.08 m and a high CCC of 0.96, indicating strong reliability. For agricultural crops, other studies reported accuracy within a similar MAE range, but they focused on maize rather than perennial fruit trees with complex canopies [5,10]. Another study [13] explored UAV-based methods and their segmentation accuracy. In comparison, the LiDAR system used in the ground demonstrated reliable canopy volume estimates with a non-significant statistical difference (*p* > 0.05) from the measured values and demonstrated effectiveness for dense orchard structures.

For the canopy volume, a *p*-value of 0.21 indicated no significant difference between the sensor-estimated and measured values, supported by the confidence interval (−0.92 to 0.26 m^3^) including zero. An underestimation bias of −0.33 m^3^ was observed, as shown in Figure 16b, but strong agreement and accuracy were demonstrated by a high CCC of 0.96 and low MAE of 0.57 m^3^, as shown in Figure 17b. These results confirm the reliability of the sensor estimates, with scope for minor refinements. A LiDAR laser scanner was employed for canopy volume estimation, demonstrating high accuracy, a fast-scanning speed, and insensitivity to light conditions. The relationship between the sensor estimated and measured results yielded r^2^ values ranging from 0.85 to 0.95 [42], consistent with the canopy volume determination efficiency observed in this study. In contrast, a study [62] reported a 5–7% error in tree canopy characterization under uneven road conditions, which slightly differed from the accuracy of the sensor-estimated canopy volume in this study. This variation may be attributed to the simulated uneven road surface conditions used in the research. Additionally, the research by Shi et al. [39] indicates that sensor-estimated row spacing was closely aligned with measured spacing, with an RMSE value of 0.019 m for corn plants and 0.07 m for apple trees. In plant phenomics, prioritizing what to measure and balancing exploratory with explanatory goals is essential [63,64].

An MLS type of LiDAR was used in [65] for the canopy density estimation of apple trees, followed by the M-estimator sample consensus (MSAC) algorithm using commercial software (The MathWorks Inc., Natick, MA, USA). The main findings of the study show that the 3D measurement algorithm was more efficient than the 2D measurement algorithm for assessing the point density of a tree canopy, and alignment during scanning was essential to avoid the error caused during experimentation. Mahmud et al. [65] scanned a total of 375 apple trees from an orchard and exhibited that more canopy points exist in the middle section of a tree compared to the top and bottom portions, and the three-dimensional algorithm is more efficient for canopy point density assessment. These findings also support this study because, for canopy volume estimation, the crown diameters were measured in the middle portion of the apple trees to obtain more dense point clouds for precise canopy estimation; this technique was also followed in this study. In the abovementioned study [65], this was emphasized by using a global positioning system (GPS) along with the canopy points in each section to obtain accurate canopy information, which was a method also followed in this study.

The results for tree spacing showed a statistically non-significant difference between the sensor estimated and measured values, with a *p*-value of 0.08 and a 95% CI of −0.18 to 0.01 for the mean difference. Despite the lack of significance, a mean bias of −0.09 m indicated consistent underestimation, as shown in Figure 16c, with a moderate agreement for a CCC of 0.84 and a moderate MAE of 0.09 m, as shown in Figure 17c. For row spacing, a *p*-value of 0.06 and CI of −0.10 to 0.002 m indicated non-significant differences, with a bias of −0.05 m suggesting underestimation, though the value was smaller than that observed for tree spacing, as shown in Figure 16d. The CCC of 0.61 and an MAE of 0.05 m, depicted in Figure 17d, demonstrated poor agreement and moderate error, highlighting the need for calibration. Overall, the sensor performed well for the tree height and canopy volume, showing high consistency and agreement, but required refinement for tree and row spacing due to moderate agreement and underestimation. A comparison of the three canopy volume estimation methods revealed no significant difference between the proposed method and the 3D alpha shape method, while the 3D convex hull method overestimated the volume by including gaps between tree leaves. The bounding box method used in this study provided accurate canopy volume estimates for the RMSE value of 0.61 m^3^ and the r^2^ value of 0.97, which supported the novelty and effectiveness of this research approach.

Many studies extensively utilized LiDAR for general vegetation mapping of cereals and forestry analysis [6,7,8], while in this study, the approach particularly involved applying 3D LiDAR technology to quantify geometric features such as the tree height, canopy volume, tree spacing, and row distance of apple trees in an orchard environment. This targeted approach addressed the unique requirements of orchard management, which are often overlooked in broader agricultural applications. The orchard-specific analysis sets this study apart from broader forestry applications, providing practical and actionable insights for precision agriculture. This study applied 3D LiDAR technology for accurately measuring the geometric features of apple trees in orchard settings. The methodology of this study emphasized extensive post-processing of point cloud data to obtain precise estimates of key parameters such as the tree height, canopy volume, and spacing. Unlike many LiDAR applications that focus on real-time data, this experimental approach prioritizes a detailed analysis to reduce noise and improve measurement accuracy. Deep learning methods were applied in a previous study [66], which relied on large datasets and UAV imagery. This study approach is simpler and more cost-effective, utilizing ROI filtering, Kd-tree search, and the RANSAC algorithm for postprocessing LiDAR data. This approach avoids the need for UAVs and extensive computational resources.

A key feature of this research is the validation of the LiDAR estimated results against ground truth measurements, which show statistically significant agreement and reliability. Advanced statistical analyses, including the concordance correlation coefficient (CCC), mean absolute error (MAE), and bias calculations, provide a solid quantitative foundation for accuracy evaluation.

While the focus was on apple orchards, the postprocessing framework might be adapted for various agricultural systems, offering flexibility for different conditions and tree structures. The practical implications of this study extend to precision orchard management, resource optimization, and sustainable farming practices. Furthermore, the research demonstrates how environmental challenges might be addressed through careful post-processing, highlighting the potential of LiDAR as a reliable tool for precision agriculture, even in suboptimal field conditions. While the accuracy of our LiDAR-estimated results yielded promising results, they are based on a relatively small sample size. As highlighted in the Results Section, the results were based on a limited dataset comprising only six measured values. Although these data points provide initial insights into geometric feature characterization performance, a broader dataset would offer measurement results with more reliable accuracy. The limited sample (six trees) constrains the generalizability of our findings and warrants caution in interpreting the accuracy of the results. Future studies should collect a larger and more diverse dataset to further validate and refine the characterization results.

## 5. Conclusions

This study demonstrated the potential of 3D LiDAR sensors for accurate geometric feature characterization of fruit trees, including tree height, canopy volume, tree spacing, and row distance in apple orchards. The study demonstrated that the sensor estimated method provided reliable results for the tree height and canopy volume, with high consistency and accuracy, as indicated by the strong agreement between the estimated and measured values with a high CCC and low MAE. However, this method showed limitations for tree and row spacing, as this consistently underestimated these parameters, suggesting the need for sensor adjustments and further refinement. The approach for canopy volume estimation highlighted the effectiveness of the bounding box method. This method yielded accurate volume estimates with a low RMSE and high r^2^, which demonstrated the potential of using this approach in precision agriculture. Although this study utilized a small dataset, the results highlight the potential of LiDAR for optimizing resource allocation and enhancing orchard productivity. Future studies with larger datasets from diverse orchard environments are recommended to refine the appropriate adjustment of sensors and expand the applicability of LiDAR technology for more precise and reliable orchard management strategies. By using a LiDAR sensor, farmers and orchard managers can make decisions to optimize resource allocation and ultimately enhance the productivity and sustainability of fruit orchards.

## Figures and Tables

**Table 1:** Technical specifications of the LiDAR sensor used in this study.

Parameter	Specifications
Operational characteristics	Number of channels: 16; range of measurement: 100 m; range accuracy: up to ±3 cm (typical); FOV (vertical): +15.0° to −15.0° (30°); horizontal FOV: 360°; vertical angular resolution: 2.0°; horizontal angular resolution (azimuth): 0.1–0.4°; rotation rate: 5–20 Hz
Laser	Class 1 eye-safe per IEC 60825-1:2007; wavelength: 903 nm
Mechanical/electrical operation	Typical power consumption of 8 W, operating voltage of 9–18 V, weight of ~830 g (without cabling and interface box), operating temperature of −10 °C to +60 °C, storage temperature of −40 °C to +105 °C
Output	Single return mode: 300,000 points/s Dual return mode: 600,000 points/s Ethernet connection: 100 Mbps, UDP packets: ToF, distance measurement, calibrated reflectivity measurement, rotation angles, synchronized time stamps (µs resolution)

Time of Flight (ToF); Field of View (FOV).

**Table 2:** Summary statistics of estimations using LiDAR sensors and measured results.

Statistical Parameters	Tree Height (m)		Canopy Volume (m^3^)		Tree Spacing (m)		Row Spacing (m)	
	Sensor	Measured	Sensor	Measured	Sensor	Measured	Sensor	Measured
Mean	3.05	3.13	13.76	14.09	3.04	3.18	3.35	3.41
S_d_	0.34	0.33	2.46	2.10	0.17	0.24	0.08	0.05
RMSE	0.09		0.61		0.12		0.07	
r^2^	0.98		0.97		0.92		0.94	
MAE	0.08		0.57		0.09		0.05	
Bias	−0.08		−0.33		−0.09		−0.05	
CCC	0.96		0.96		0.84		0.61	
t-statistic	−3.49		−1.44		−2.19		−2.47	
*p*-value	0.02		0.21		0.08		0.06	
CI	(−0.14, −0.02)		(−0.92, 0.26)		(−0.18, 0.01)		(−0.10, 0.002)	

S_d_ is standard deviation. RMSE is root mean square error. r^2^ is coefficient of determination. MAE indicates mean absolute error. Bias indicates bias (mean of differences). CCC is concordance correlation coefficient. CI is confidence interval for mean difference.

## Data Availability

Data are contained within the article.
